# Management of Thyroid nodules in adult patients

**DOI:** 10.1186/1758-3284-2-11

**Published:** 2010-05-05

**Authors:** Chee Yean Eng, Muhammad S Quraishi, Patrick J Bradley

**Affiliations:** 1 ENT Department, Doncaster Royal Infirmary, Armthorpe Road, Doncaster DN2 5LT, UK; 2 Queen's Medical Centre, Derby Road, Nottingham NG7 2UH, UK

## Abstract

Thyroid nodule is a common presentation and requires a structured diagnostic approach to ascertain the risk of malignancy and determine appropriate management. This review article highlights the key points in the history and examination which can help with risk stratification. It also discussed the application of fine needle aspiration cytology findings and the British Thyroid Association Guidelines in clinical practice.

## Introduction

Although thyroid nodule is a common presentation, thyroid cancer is rare. Thyroid nodules can be detected by palpation in 10% of women and 2% of men [1]. The prevalence of thyroid nodules can be 50% or more if ultrasonography was used [1]. On the other hand, the annual incidence of thyroid cancer in the UK was reported at 3.5 per 100,000 women and 1.3 per 100,000 men [1]. Thyroid cancer is the most common endocrine malignancy but only represents 1% of all malignancies [1]. It is therefore crucial to have a clear diagnostic approach to ensure patients presenting with thyroid nodules are managed appropriately and are not over or under-treated. Thyroid cancer has a favourable prognosis and accounts for less than 0.5% of cancer deaths [2].

## Clinical assessment

It is important to obtain a good history focusing on the gender (male has a higher risk) and age at presentation should be noted. The risk of malignancy is increased for thyroid swelling in patients less than 16 years old and above 45 years old. It should include the duration of the thyroid swelling and, more importantly, the rate at which it is growing. History of neck irradiation and family history of thyroid cancer should be noted. Other associated symptoms such as difficulty swallowing or breathing would suggest compressive effect from the thyroid swelling. A hoarse voice is a strong indication of recurrent laryngeal nerve palsy and malignancy [3,4]. These are summarized in Table 1.

**Table 1:** Risk factors for thyroid malignancy. Baseline UK annual incidence for thyroid cancer: 2 - 3/100,000 population [3].

Risk factors	Risk of malignancy
Gender [4,9]	Male: 2 - 3 times increased risk.

Age [4,8]	Less than 20: Risk of malignancy doubled. Above age 45: Increased risk of malignancy. Above 70: Risk of malignancy quadrupled.

Ionising radiation [3,10]	Latency period is usually 10 - 15 years and mostly occurs 20 - 30 years after exposure.
	There is a 40% absolute risk of malignancy for a thyroid nodule in a patient with previous radiation exposure [9].
	Low dose: 100 times increase risk of malignancy (lifetime risk).
	High dose: 300 times increase risk of malignancy (lifetime risk).

Family history [3]	Presence of thyroid cancer in family members increases risk of malignancy.

Tumour size [4,11]	The larger the tumour size, especially when >4 cm, or the presence of obstructive symptoms indicates higher risk of malignancy.

Rate of growth [3,10,11]	History of rapid growth in a few weeks indicates higher risk of malignancy.

Hoarse voice or vocal cord palsy with recurrent laryngeal nerve involvement [11]	Presence of hoarse voice or vocal cord palsy indicates high risk of malignancy.

Cervical lymphadenopathy [11]	Presence of cervical lymphadenopathy indicates high risk of malignancy.

Characteristics of thyroid swelling [11]	Firm/hard consistency or fixed swelling indicates high risk of malignancy. Soft, mobile or cystic swelling indicates low risk of malignancy.

Examination of the neck should include palpation of the thyroid gland to determine the characteristics (size, consistency, mobility, cystic, single nodule, multinodular, or dominant nodule in multinodular goiter) and for any palpable cervical lymphadenopathy. A note should be made about voice quality and if in any doubt, a clinic flexible laryngoscopy to directly visualise and assess the movement of the vocal cords could be performed. These are summarized in Table 1.

Patients with difficulty breathing (increase respiratory rate or decrease oxygen saturation) or stridor should be referred as an emergency and be seen on the same day.

### Serological investigations

Routine Thyroid Function Test (TFT) including Thyroid Stimulating Hormone (TSH) should be performed to determine the patient's thyroid status. T3 and T4 levels will be required if TSH level was abnormal. When hypothyroidism is confirmed, Thyroid Peroxidase Antibodies (TPA) should be requested to check for auto-immune thyroid disease such as Hashimoto's thyroiditis. Thyroglobulin level does not help in the initial management of thyroid nodule and therefore is not recommended.

Basal plasma calcitonin levels may be useful if Medullary Thyroid cancer (MTC) is suspected such as when there is a family history of medullary thyroid cancer or paraneoplastic syndromes. These may include Cushing's syndrome (ACTH) or carcinoid syndrome with watery diarrhoea and vasomotor flushing. Clinician should also be vigilant for pheochromocytoma which is associated with MTC in Multiple Endocrine Neoplasia (MEN) Type II. Patients with pheochromocytoma can present with sympathetic nervous system hyperactivity such as anxiety, palpitations, tremor, hypertension and cardiac arrhythmias.

### Biopsy and Imaging

Fine needle aspiration cytology (FNAC) is the most important step in the management of thyroid nodules. FNAC has a sensitivity ranging from 65% - 98% and specificity ranging from 72% - 100% [5]. The false positive rate for cancer varies from 0 - 7% and the false negative rate for cancer varies from 1% - 11% [5]. FNAC can be performed free-hand or ultrasound-guided to increase confidence if the lesion is palpable.

Ultrasound-guided FNAC is gaining popularity and has been found to improve accuracy of FNAC. The acellular or non-diagnostic (Thy 1) aspirate is reduced from 14% in free-hand FNAC to 8% in ultrasound-guided FNAC [5]. The sensitivity (from 92% to 98%) and specificity (from 69% to 71%) of FNAC was also improved with ultrasound guidance [6]. In addition, ultrasound-guided FNAC can be used to help localise impalpable lesion, lesions less than 1 cm, or when initial free-hand FNAC was non-diagnostic.

Core biopsy (with or without ultrasound guidance) should be considered after two aspiration procedures showing non-diagnostic specimen (Thy 1) or when a thyroid lymphoma was suspected. Thyroid lymphoma typically presents with a rapidly increasing neck swelling in an elderly woman or on a background of autoimmune thyroiditis.

**Figure 1:**
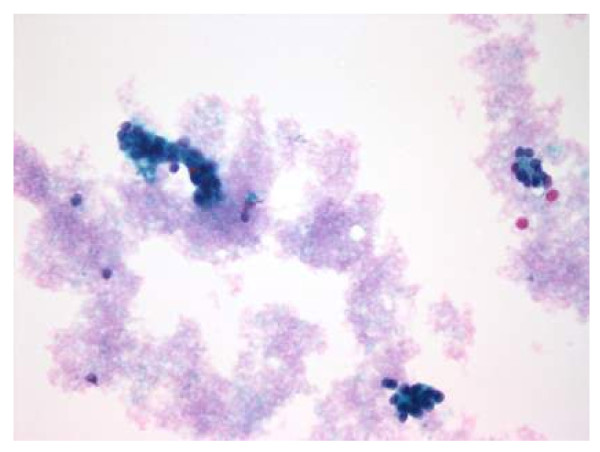
**Thyroid follicular cells in a microfollicular pattern (Thy 3)**.

**Figure 2:**
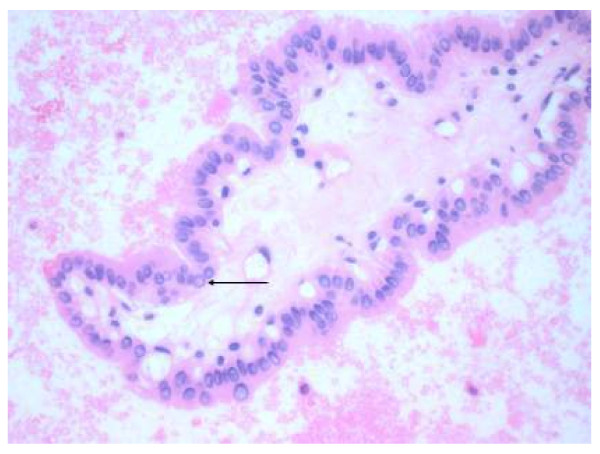
**Cytology of a Papillary Thyroid carcinoma**. Showing the fibrovascular core and an intranuclear inclusion (arrowed).

Cytology results can be placed in 5 diagnostic categories (Thy 1 - Thy 5) as indicated by the British Thyroid Association guidelines [1]. This will help with subsequent management and is summarised in Table 2 (Figure 1 and 2) below:-

**Table 2:** FNAC Diagnostic categories and recommended actions

Diagnostic category	Description	Recommended action
Thy 1	Non-diagnostic, insufficient sample. Cyst containing colloid or histiocytes only, in the absence of epithelial cells.	To repeat FNAC. Ultrasound-guidance may help. If cyst aspirated to dryness with no residual swelling, clinical/ultrasound follow-up alone may be sufficient.

Thy 2	Benign, non-neoplastic. Cyst containing benign epithelial cells.	Repeat FNAC in 3 - 6 month. Two non-neoplastic results 3 - 6 months apart should exclude neoplasia.

Thy 3	Follicular or Hurthle cell lesion/suspected follicular or Hurthle neoplasm. (Figure 1)	MDT discussion - diagnostic lobectomy.

Thy 4	Suspicious of malignancy. (Figure 2)	MDT discussion - surgical intervention, e.g. Total thyroidectomy.

Thy 5	Diagnostic of malignancy.	MDT discussion - surgical intervention, e.g. Total thyroidectomy.

**Figure 3:**
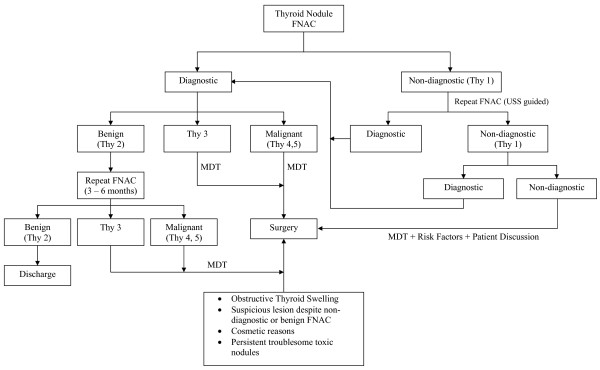
**Thyroid Nodule management flow chart**.

Given the above false-negative rate of FNAC results, the probability of a benign thyroid nodule being accurately diagnosed as benign from a single FNAC is 90%. However, the accuracy of diagnosis increases significantly to 98% if two separate aspirates were performed on separate occasions [7]. As such, having 2 aspirates decreases the false negative rate to 1.2% [7]. A diagnostic flowchart is shown in Figure 3.

**Figure 4:**
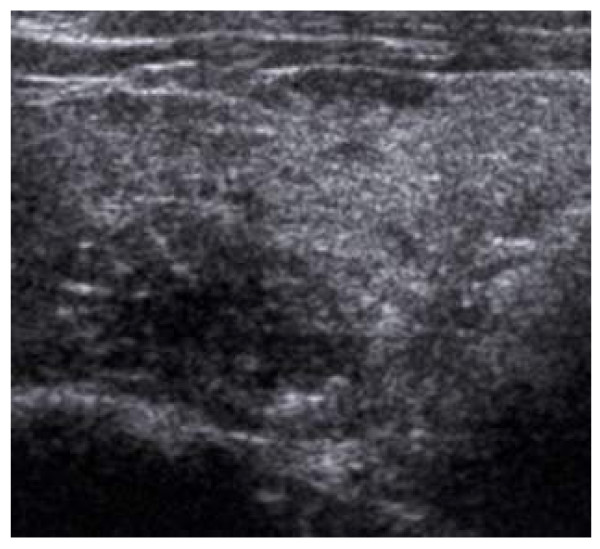
**Ultrasound features: Micro-calcification - histology proven papillary carcinoma**. Hypoechoic, ill-defined margin, internal microcalcifications.

Ultrasound can be used to accurately document the size of thyroid swelling and therefore serial scans allow better assessment of growth. Certain ultrasound characteristics on thyroid swelling have been shown to be associated with higher or lower risk of malignancy. Comet tail sign and coarse calcification suggests very low risk of malignancy. Hypoechoicity and absent halo with indistinct margin are associated with moderate risk of malignancy. The presence of microcalcification (Figure 4) is highly suggestive of malignancy and especially papillary carcinoma [4,7].

Computed Tomography (CT) and Magnetic Resonance Imaging (MRI) are necessary in some cases to determine the staging and extent of the disease, and in planning surgery. CT and/or MRI is indicated when there is presence of a fixed thyroid mass or patients with haemoptysis indicating potential involvement of surrounding structures. Other important indications include cervical lymphadenopathy or when limits of the goiter cannot be determined clinically such as retrosternal goitre. CT or MRI can demonstrate involvement of the larynx, pharynx, trachea, oesophagus or major blood vessels. CT can also help in detecting pulmonary metastasis. It is important to avoid iodine-contrast media in CT scan to ensure subsequent radioiodine treatment uptake by remaining thyroid tissue is not compromised. This difficulty may be overcome by requesting for Gadolinium-enhanced MRI scan.

### Euthyroid clinically palpable solitary nodule or nodule > 1 cm

These patients have the highest risk of thyroid cancer for all patients presenting with thyroid nodules (up to 20% will be malignant) [3]. They should be carefully evaluated and managed in accordance with the results of fine needle aspiration cytology.

In high risk clinical group (Table 1) or lesions with suspicious ultrasound characteristics previously described, despite benign FNAC (Thy 2), diagnostic lobectomy may sometimes be appropriate after MDT discussion.

Follicular and Hurthle cell lesions are commonly associated with Thy 3 Classification. This is because these 2 lesions can only be differentiated by the presence or absence of capsular, vascular or lymphatic invasion on histological examination. Furthermore, these lesions have been noted to have a malignancy rate of 10% - 30% [8]. Surgical management of these lesions is usually indicated after MDT discussion.

### Thyroid nodule associated with Hypo/Hyper-thyroidism

These nodules are very unlikely to be cancer. They are more likely to be benign toxic nodule or Hashimoto's thyroiditis. The frequency of malignancy in cold nodules is 10 - 20% and only 4% in hot nodules [3,9].

These nodules should still be aspirated and if confirmed to be benign (Thy 2) after 2 aspirates 3 - 6 months apart, with no other suspicious features, can be safely managed by an endocrinologist. These patients can be referred back for re-evaluation if there was any change in the swelling.

### Thyroid cystic swelling

Thyroid cyst should be clearly stated to help pathologist in interpreting FNAC. Cyst should be aspirated to dryness and it is important to note whether there is any residual mass. Any residual mass should be immediately aspirated and send as separate specimen [1].

For thyroid cyst that is shown to be benign on FNAC and does not recur at follow-up, clinical observation alone may be sufficient.

Recurrent thyroid cyst should be re-aspirated during follow-up and sample sent for cytological examination. Patients with high risk factors in history and examination can be considered for diagnostic lobectomy. Some surgeons would consider diagnostic lobectomy for cyst that has recurred for 3 times or more (anecdotal evidence). Surgery can also be considered at patient's request.

### Dominant nodule in multinodular goiter

Patients with hyper- or hypothyroidism associated with multinodular goiter with no other suspicious features in history and clinical examination have a low risk of thyroid cancer^1^. These patients are routinely referred to an endocrinologist.

When a dominant nodule is noted to be growing and become suspicious, it should be aspirated and treated accordingly depending on cytology results.

Low-risk patients who are euthyroid with multinodular goiter that has been present for years, and has not changed in size, have a very low risk of thyroid cancer [1]. These patients can be observed at intermediate or long intervals.

### Clinically non-palpable incidental thyroid swelling < 1 cm ('Incidentalloma')

These are not uncommonly noted during surgery or imaging performed for another purpose. In patients with low risk characteristics (as per history, examination, USS findings), these nodules have very low risk of cancer [1]. In addition, there is no evidence to show that treatment of subcentimeter microcarcinomas improves outcome [2,9].

The exception to the above is an 'incidentalloma' identified by FDG-PET scan. It was shown that these carry a 50% chance of malignancy and therefore should be managed as per a solitary thyroid nodule [9]. Likewise, 'Incidentalloma' larger than 1 cm should also be managed as per a solitary thyroid nodule.

## Conclusion

Thyroid nodule is a common clinical presentation and it is important to have clear diagnostic framework to ensure appropriate management of these patients. Although fine needle aspiration cytology forms the cornerstone of diagnosis, good history and examination will help stratify the risk of malignancy and ultimately the best management option chosen.

## Competing interests

The authors declare that they have no competing interests.

## Authors' contributions

CYE drafted the manuscript and the diagnostic flow-chart.

MSQ obtained clinical photos, coordinated, and drafted the manuscript.

PJB conceived the approach to the subject of this manuscript and proofread the manuscript.

All authors read and approved the final manuscript.
